# Impact of Vitamin D Supplementation on Inflammatory Markers’ Levels in Obese Patients

**DOI:** 10.3390/cimb43030114

**Published:** 2021-10-14

**Authors:** Michał Wiciński, Mateusz Ozorowski, Eryk Wódkiewicz, Stephan Walter Otto, Karol Kubiak, Bartosz Malinowski

**Affiliations:** 1 Department of Pharmacology and Therapeutics, Faculty of Medicine, Collegium Medicum in Bydgoszcz, Nicolaus Copernicus University, M. Curie 9, 85-090 Bydgoszcz, Poland; michal.wicinski@cm.umk.pl (M.W.); mateusz-ozorowski@o2.pl (M.O.); bartosz.malinowski@cm.umk.pl (B.M.); 2 Department of Urology, Raphaelsklinik, 48143 Münster, Germany; stephotto@gmx.de; 3 Department of Obstetrics and Gynecology, St. Franziskus-Hospital, 48145 Münster, Germany; karolkubiak85@googlemail.com

**Keywords:** vitamin D, inflammation, obesity, pathways, pharmacology

## Abstract

In view of research suggesting a possible beneficial impact of vitamin D on systemic inflammatory response, the authors decided to investigate an influence of vitamin D supplementation on serum levels of certain inflammatory markers in obese patients. The current study included such biomarkers as interleukin-6 (IL-6), pituitary adenylate cyclase-activating peptide (PACAP), advanced oxidation protein products (AOPP), C-X3-C Motif Chemokine Ligand 1 (CX3CL1), monocyte chemoattractant protein-1 (MCP-1), and nitric oxide (NO). The measurements were performed with the ELISA method before and after 3-month-long supplementation of 2000 IU of vitamin D orally. The results showed that the therapy did not induce any statistically significant changes in serum levels of MCP-1, IL-6, CX3CL1, and PACAP. The supplementation was related to a significant increase in measurements of NO and AOPP levels, although the correlation analysis between vitamin D concentration after its supplementation and the concentration of the molecular parameters did not show significant relation. In conclusion, our study seems to contradict certain aspects of findings available in the literature regarding the vitamin D’s impact.

## 1. Introduction

Obesity is the most common risk factor for cardiovascular disorders and is directly connected with morbidity and mortality. Scientists suggest that adipose tissue is not only an energy deposit but that it has also a secretory function. Adipocytokines such as IL-6, TNFα, and MCP-1 are involved in inflammatory processes and belong to pathophysiological risk factors of various cardiovascular disorders. Moreover, it has been found that they may play an important role in cancer development and even dementia. Currently, ongoing experiments throw a new light on the obesity-linked clinical risk factors [1].

### 1.1. Obesity and Inflammation

We can divide adipose tissue into three types: white, brown, and pink adipose tissue. During pregnancy, as well as lactation and post-lactation periods, subcutaneous white adipocytes convert to milk-producing glands formed by lipid-rich elements that can be defined as pink adipocytes [2]. The brown type, although especially abundant in newborns and hibernating mammals, remains present and metabolically active in adult humans. Its primary function is thermoregulation. What is more, it has been proven to be inversely corelated with BMI in adults [3]. Recent studies suggest two types of thermogenic adipocytes with distinct developmental and anatomical features: classical brown adipocytes and beige adipocytes. The latter have recently attracted special interest because of their ability to dissipate energy and potential to differentiate themselves from white adipocytes. Numerous factors affecting the differentiation process have become attractive therapeutic targets for treatment of obesity and obesity-related diseases (reviewed in [4]). White adipose tissue is known to be involved in production and regulation of various substances involved in the inflammation and immune response. Monocytes, macrophages, adiponectin, endothelial cells, lymphocytes, and a cross talk between them play a crucial role in those processes. Adipose tissue can be called a double-edge sword in the context of our physiology and health state. It produces pro- and anti-inflammatory molecules [5]. Many clinical studies suggest that overexpression of pro-inflammatory mediators in obese patients is the result of adipocyte hypertrophy, which leads to hypoxia and macrophage infiltrations [6]. Accumulation of non-esterified fatty acids (NEFA) increases the activity of c-Jun N-terminal kinases, protein kinase R, and Toll-like receptors (TLR) [7]. Those molecules are implicated in interleukin-6 (IL-6) secretion, which leads to hepatocyte-dependent C-reactive protein (CRP) production. This systemic inflammatory response determines platelet and white blood cells’ release [8]. The process of chemotaxis, that is, migration of morphological elements of the blood, is mediated through the expression of various molecular agents. Among them are CC chemokines, a family of monocyte and macrophage chemoattractants. The C-C chemokine receptor type 2 (CCR2) was found to be increased in obesity [9]. Scientists from the University of Central Florida reported that a novel zinger finger protein called MCPIP (MCP-1-induced protein) proved to have a potential to induce adipogenesis without PPARγ participation. It has been found that forced expression of MCPIP induces expression of the C/EBP family of transcription factors and adipogenesis in PPARγ (−/−) mouse embryonic fibroblasts [10]. All the findings may suggest that higher MCP-1 concentration is the result of adipose tissue excess, possibly due to macrophage infiltration. It is noteworthy that MCP-1 signaling pathways may develop obesity by themselves [11]. Fractalkine and its receptors (CX3CL1/ CX3CR1) are known to be essential mediators in monocyte/macrophage cell migration [12]. Nagashimada et al. found that CX3CL1–CX3CR1 signaling participates in adipose tissue inflammation and insulin resistance development in obese mice [13]. As another subfamily of molecular attractants, the CXC chemokines act mainly on neutrophils and lymphocytes, whereas the CC chemokines, such as the aforementioned MCP-1/CCL2, act majorly on monocytes and lymphocytes without affecting neutrophils [14]. Tumor necrosis factor α (TNFα) is highly expressed in fat tissue [15]. Its concentrations correlate with a degree of obesity. The stromal vascular cells are mainly responsible for TNF-α secretion in humans. It should be mentioned that there are two forms of soluble TNF α receptors: type 1 and type 2. Both types can be found in adipose tissue; however, type 1 shows higher expression in omental than subcutaneous adipocytes [16]. Pituitary adenylate cyclase-activating polypeptide (PACAP) is involved in inflammatory process as well as glucose and lipid metabolism [17]. Xiao et al. showed that the Fas apoptosis inhibitory molecule (FAIM) can be a new mediator for Akt2 pathways. What is more, PACAP may promote FAIM expression in hepatocytes, leading to a consequent decrease in body mass. Chinese scientists proved that FAIM may reduce adipogenesis proteins such as sterol regulatory element-binding protein 1 (SREBP1), sterol regulatory element-binding protein 2 (SREBP2), stearoyl-CoA desaturase (SCD1), and fatty acid synthase (Fas) [18]. SREBP1c (sterol regulatory element-binding protein 1c) is a metabolic syndrome-associated transcription factor controlling fatty acid biosynthesis related to glucose/insulin stimulation. Oxidative stress increases lipid accumulation, which subsequently promotes the generation of reactive oxygen species (ROS). ROS may stimulate lipid accumulation in HepG2 cells via SREBP1c activation leading to an example of a vicious circle [19]. Based on the facts, it is possible that advanced oxidation protein product (AOPP) constitutes an adequate marker for evaluation of such phenomena [20]. Nitric oxide (NO) is considered a free radical of limited bioactivity. Due to its half-life of 2 ms to 42 s, the potential significance of NO action seems to be dependent on the availability of other reacting molecules [21]. Apart from the oxidative potential, NO has multiple biological properties leading to changes in, among others, angiogenesis, platelet aggregation, leukocytes interactions, synaptic transmission, and immune system signaling on various levels [22,23]. Formerly known as endothelial-derived relaxing factor (EDRF), nitric oxide is one of the most potent regulators of blood flow acting via soluble guanylyl cyclase (sGC) activation and cyclic guanosine monophosphate (cGMP) production-related vasodilatation [24,25]. Nitric oxide synthase (eNOS) is an enzyme involved in the synthesis of nitric oxide. There are a few isoforms of eNOS implicated to various extents in different processes including regulation of blood pressure, regulation on medullar and hypothalamus level (eNOS3), penile erection (eNOS1), microvascular circulation (both eNOS1 and eNOS3), or inflammatory processes (eNOS2) [26,27,28]. There are findings showing that NO bioavailability is decreased in obese and diabetic states. The data concern both animal models of obesity and diabetes [29,30] as well as obese and diabetic human patients [31,32]. The disruption in balance between its generation and degradation may be caused by changes in expression of eNOS, impairments in eNOS activity, or some influence of related factors [23]. Long-term elevation in NO levels results in mitochondrial biogenesis [33,34], directing cell programming towards higher metabolic capacity. 

### 1.2. Vitamin D: Structure and Function

The popularity of vitamin D increased significantly in the last decade. Supplementation of vitamin D is constantly tested, with more and more reports of its beneficial effect not only in the calcium–phosphate turnover, but also on skeletal, immune, and cardiovascular systems. The term “vitamin D” describes two compounds: ergocalciferol (Vitamin D2 found in plants) and cholecalciferol (vitamin D3 found in animals) [35]. The last form is synthesized in the skin with the participation of UV-B radiation. Activity of vitamin D2 and vitamin D3 is comparable. Vitamin D and its metabolites are soluble in fats [36]. Metabolism of vitamin D starts in the liver with the hydroxylation at C25. This reaction results in the first metabolite: 25(OH)D3, calcifediol. The half-life of 25(OH)D3 is approximately 21 days. It does not fluctuate rapidly; therefore, calcifediol is the best marker of vitamin D supply independently of the source (skin synthesis or diet) [37]. Kidneys play a crucial role in vitamin D metabolism. At this spot, hydroxylation at C1 takes place and 1,25(OH)2D3 is formed. This most active type of vitamin D has a short half-life of 1 to 7 h [38]. Calcitriol is structurally related to steroids and it shows hormone-like effects. It affects targeted cells via vitamin D receptors (VDR) in the nucleus. Those receptors are located in many organs such as the heart, brain, blood vessels, adrenal gland, pancreas, small and large intestines, and others [39]. Calcitriol may regulate transcription of over a few hundred genes; thus, the presence of VDR is connected with the wide spectrum of its action on numerous cell types. The first factor that determines the mechanism of action of vitamin D is the binding potential to VDBP (Vitamin D-binding protein). Within the all-natural form of vitamin D found in serum, calcifediol shows the highest affinity [40]. Overexpression of VDBP in our physiological compartments helps in many pathological states, i.e., liver injury or nephrotic syndrome. Moreover, megalin is the receptor found in proximal tubule, which reabsorbs both VDBP and RBP (retinol-binding protein). It has been reported that megalin can bind the skip protein, the crucial activator of VDR. Intracellular VDBP controls the transfer of 25(OH)D3 to mitochondria (IDBP3) and calcitriol binding to the VDR (IDBP1). After binding to a ligand, VDR is able to create a complex with retinoid X receptor: VDR/RXR [41]. The whole complex is then linked to vitamin D responsive elements (VDRE), that is, DNA sequences found in the promoter region of vitamin D-regulated genes [42].

### 1.3. Vitamin D and Inflammation

It has been reported that vitamin D may play a crucial role in the inflammatory process. The molecular basis to that statement is the presence of VDRs in macrophages, neutrophils, activated lymphocytes B and T, and dendritic cells [43]. Macrophages’ stimulation of Toll-like receptors results in upregulation of VDR and CYP27B1 (the gene that encodes the 25-hydroxy vitamin D-1α-hydroxylase) [44]. Overexpressed CYP27B1 leads to accumulation of 1,25(OH2) D3 and subsequent target gene transcription via VDRE. What is more, Toll-like receptor pathways can be regulated by suppression of cytokine signaling 1 protein (SOCS1) [45]. Numerous studies suggest that vitamin D inhibits IL-6 and TNF-α production via the histone H4-dependent manner in human monocytes [46]. Other trials showed that vitamin D may decrease MCP-1 and IL-6 release due to reduced activation of NF-κB in macrophages. The 1,25(OH)2D3 affects differentiation of human dendritic cells (DCs), probably linked to the limitation of their surface expression for CD40 and CD80 [47]. Scientists from the University of Birmingham found that vitamin D inhibits production of proinflammatory mediators such as IFN-γ, IL-17, and IL-21 and promoted the development of T-reg cells. The last ones may also stimulate vitamin D metabolism via the aforementioned upregulations of CYP27B1 [48]. Endothelial nitric oxide synthase (eNOS) expression was reduced in mice lacking the VDR gene. Additionally, increased endothelin-1 (ET-1) expression and sensitivity to the angiotensin II hypertensinogenic properties were observed in the same study [49]. Tare et al. noticed that mesenteric arteries of rats with vitamin D deficiency were characterized by a 2-fold decrease in the ability to relax. The mechanism of impairment has been related to NO and endothelial hyperpolarizing factor (EDHF) signaling [50]. It is possible that the abovementioned effects could be reversed by vitamin D supplementation [49]. Studies of Andrukhova et al. showed that cholecalciferol is a direct transcriptional regulator of eNOS [51]. 

**Figure 1:**
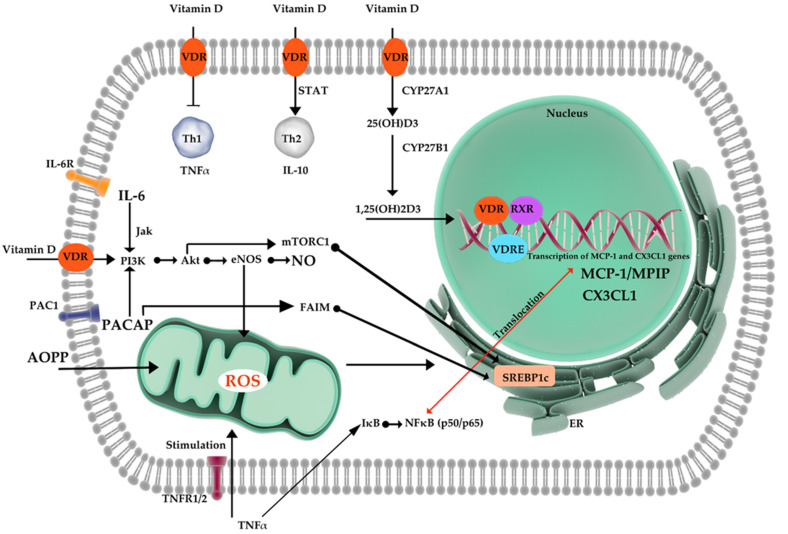
Potential mechanisms of vitamin D’s impact on inflammatory markers. Abbreviations: TNF-α, tumor necrosis factor α; TNFR1/2, TNF receptor-1/ TNF receptor-2; IκB-, the IκB kinase’; NFκB, nuclear factor kappa-light-chain-enhancer of activated B cells; SREBP1c, Sterol regulatory element-binding transcription factor 1c; 1,25(OH)2D3, calcitriol/1,25-dihydroxycholecalciferol; 25(OH)D3, Calcifediol/calcidiol; CYP27B1, cytochrome P450 family 27 subfamily B member 1; VDR, Vitamin D Receptor; VDRE, Vitamin D response element, RXR, retinoid X receptor; MCP-1, monocyte chemoattractant protein-1; CXCL1, chemokine (C-X-C motif) ligand 1; Th1, type 1 T helper cells; Th2, type 2 T helper cells; IL-10-, Interleukin 10; IL-6, Interleukin 6, IL-6R, Interleukin 6 receptor; PACAP, Pituitary adenylate cyclase-activating peptide; PAC1, activated GPIIb/IIIa complex; AOPP, Advanced oxidation protein products; Jak- Janus-activated kinases; PI3K/Akt, The phosphatidylinositol 3-kinase (PI3K)/protein kinase B (AKT) signaling pathway; mTORC1, mammalian target of rapamycin complex 1; eNOS, Endothelial Nitric Oxide Synthase; NO, nitric oxide; FAIM, Fas apoptotic inhibitory molecule 1.

In view of the above, we decided to investigate the influence of vitamin D supplementation on serum levels of certain inflammatory markers in obese patients. A summary of the proposed mechanism underlying a potential influence of vitamin D on inflammatory markers levels is presented in Figure 1.

## 2. Materials and Methods

The study group included 33 obese patients, 16 women and 17 men. Participants signed Written, Informed Consent Form at the beginning of the study. Those patients were assigned to a control group (Time point 0, before supplementation of vitamin D) or intervention group (Time point 1, after 3 months of vitamin D supplementation). Inclusion criteria were set according to anthropometric measurements such as body weight, BMI, and age between 23–71 years old. Moreover, all participants did not supplement vitamin D before the start of the experiment and showed vitamin D deficiency. The study design was approved by the ethics committee of Collegium Medicum in Bydgoszcz, Nicolaus Copernicus University, in Toruń (approval number KB48/2019). The study was conducted according to the criteria set by the declaration of Helsinki and each subject signed an informed consent before participating in the study.

### 2.1. Incusion and Exclusion Citeria

Inclusion criteria for the study were age of over 18 years old and obesity (by BMI or % body fat). Criteria excluding participation in the study were nicotinism, use of estrogen or estrogen–progestogen-based hormone therapy, myocardial infarction or stroke within the last year, neoplastic diseases, dialysis, liver diseases, osteoporosis, pregnant women, possible vitamin D malabsorption (cystic fibrosis, Crohn disease), allergy to the ingredients contained in a cholecalciferol preparation tablet intended for patients, or refusal to collect blood for testing.

### 2.2. Measurements

Anthropometric characteristics were provided on an In-body device in the Department of Pharmacology and Therapeutics, Medicine Faculty, Collegium Medicum in Bydgoszcz. Vitamin D was administrated at a dose of 2000 IU. The concentration of vitamin D at all stages of the experiment was measured on a Beckman Coulter DXI 800 by the chemiluminescence method (mini Vidas Blue 25 H Vitamin D total quantitative kit). Biomarkers were determined with the ELISA method on a BioTek EPOCH Instrument using Elisa Kits by SunRed for such factors as PACAP (catalog number: 201-12-1308), AOPP (catalog number: 201-12-1267), CX3CL1 (catalog number: 201-12-2102), MCP-1 (catalog number: 201-12-0125), NO (catalog number: 201-12-1511), and Elisa Kits by DRG (catalog number EIA-4640) for IL-6. Body mass composition was determined with an InBody Composition 270 analyzer (four-limb leads’ electrodes). Distribution of abnormal body mass stages was determined according to WHO recommendations (Table 1).

### 2.3. Statistical Analysis

Quantitative results were presented as mean values with standard error of the mean (±SEM) and additional minimum and maximum values. The compliance of the results’ distribution with the normal distribution was checked using the Shapiro–Wilk test separately for the results obtained before (Time point 0) and after the 3-month vitamin D supplementation (Time point 1). The comparison of the results having a normal distribution for the dependent variables was provided using the Student’s t test for the dependent variables and, when the variables were not normally distributed, using the Wilcoxon test. The third type of test was a multivariate regression analysis. The dependent variables (dependent, Y) were the parameter of vitamin D concentration before supplementation and after the 3-month supplementation. The explanatory variables (independent, x) were the parameters of anthropometric features and the analyzed molecular markers. Multivariate regression analyses were performed using the method of progressive stepwise introduction to the model with the adopted testing level at the level of F > 1.0. The redundancy/collinearity of the variables in the model was assessed using the wedge-shaped methods (cross-correlation table for all parameters used) and, finally, the model used a preventive algorithm in the form of ridge regression. Statistically significant differences between the groups were considered as a *p* value < 0.05. Correlated variables were considered when R value was > 0.3 with simultaneous *p* value < 0.05 for the statistical test. The interpretation of the correlation index is included in Table 1. In multiple regression models, where there was a final model fit index r, the results of the interpretation are also presented in Table 2. All calculations were provided via GraphPad Prism 8.0.

## 3. Results

The baseline characteristics include age, gender, weight, height, BMI, adipose tissue mass, skeletal muscles mass, and visceral fat levels. Body weight and BMI values did not differ significantly before and after the therapy (Table 3).

According to the World Health Organization interpretation of the BMI values, all subjects were obese to varying degrees. At the same time, this observation was also true for the interpretation of the BMI values after the therapy. After the end of the 3 months of vitamin D supplementation, the value of this index did not change significantly. Among the individuals, both before and after vitamin D supplementation, the majority were patients with first-degree obesity (before: 39.39%; after: 36.36%) and second-degree obesity (before: 36.36%; after: 39.39%). After the therapy, the percentage of people with first-degree obesity (39.39% vs. 36.36%, before vs. after) and third-degree (21.21% vs. 18.18%, before vs. after) slightly decreased. With second-degree obesity (36.36% vs. 39.39%, before vs. after) and overweight (3.03% vs. 6.06%, before vs. after) it slightly increased. The anthropometric features assessed before and after vitamin D supplementation also included skeletal muscle mass, adipose tissue mass, % of adipose tissue, and the level of visceral fat (Table 4).

Results for any of the four characteristics listed (skeletal muscle mass, adipose tissue mass, % of adipose tissue, and visceral fat levels) did not show significant differences between the time points 0 and 1. The concentration of vitamin D 25-(OH) in all subjects before the therapy equaled, accordingly, an average level of 18.22 ± 1.103 ng/mL, and after the therapy it increased to the level of 29.89 ± 1.160 ng/mL. The Wilcoxon pair test was statistically significant (*p* < 0.001).

In Table 5 and Table 6, divided results for women and men are presented. In Table 7 we have shown Vitamin D concentration before and after the 3-month supplementation.

In the next step of our experiment, molecular parameters involved in the inflammatory process were determined. Our measurements included interleukin-6 (IL-6), Pituitary adenylate cyclase-activating peptide (PACAP), Advanced oxidation protein products (AOPP), C-X3-C Motif Chemokine Ligand 1 (CX3CL1), Monocyte Chemoattractant Protein-1 (MCP-1), and nitric oxide (NO). All parameters were compared between two time points (Time point 0 vs. time point 1). MCP-1, IL-6, CX3CL1, and PACAP did not show significant differences before and after vitamin D therapy. Results have been presented in Table 8.

The AOPP concentration in the subjects before the therapy reached the average level of 55.07 ± 12.21 nmoL/mL, and after the therapy it increased to the level of 58.94 ± 12.10 nmoL/mL. The difference between the mean concentrations of AOPP before and after treatment (3.87), as shown by the Wilcoxon pair test, was statistically significant (*p* = 0.047). The concentration of NO in all subjects before the therapy reached the average level of 39.19 ± 10.96 µmoL/L, and after the therapy it increased to the level of 70.02 ± 13.80 µmoL/L. The difference between the mean concentrations of NO before and after treatment (30.83) µmoL/L), as shown by the Wilcoxon pair test, was statistically significant (*p* = 0.021).

The Spearman r correlation analysis between vitamin D concentration before its supplementation and the concentration of molecular parameters (MCP1, IL-6, NO, CX3CL1, AOPP, and PACAP) before Vitamin D therapy did not show any significant relations between the studied values (Table 9).

The Spearman r correlation analysis between vitamin D concentration after its supplementation and the concentration of molecular parameters (MCP1, IL-6, NO, CX3CL1, AOPP, and PACAP) after the 3-month Vitamin D therapy did not show any significant relations between the studied values (Table 10).

## 4. Discussion

Nitric oxide’s modulatory influence on vascular and immune function is beyond discussion. Vast experimental evidence presents NO as a potentially protective agent as well as an unbeneficial one, related to oxidative injuries [52,53]. The nature of vitamin D’s impact on NO signaling remains controversial. Calcitriol is known to inhibit LPS-induced immune activation in human endothelial cells [54]. Activation of 1α-hydroxylase in macrophages elevates the level of calcitriol, which inhibits the iNOS expression and reduces NO production within LPS-stimulated macrophages [55]. In that case, calcitriol production by macrophages may constitute a protective mechanism against the oxidative injuries that are caused by the so-called NO burst. The same effect on iNOS has been observed in rat CNS during an experimental model of allergic encephalomyelitis [56]. Conversely, according to Andrukhova et. al., vitamin D has a potential to improve endothelial function and health by an increase in signaling for the transcription of endothelial nitric oxide synthase (eNOS) [50]. Studies in mice have proven that subjects deprived of the eNOS and/or nNOS gene exhibit metabolic syndrome, leading to possible vascular consequences [57,58].

In the current investigation, NO concentrations in the studied patients reached an average level of 39.19 ± 10.96 µmoL/L prior to vitamin D supplementation. Subsequent therapy increased its levels to 70.02 ± 13.80 µmoL/L in a statistically significant manner (*p* = 0.021). Previous studies have shown that vitamin D may upregulate eNOS expression and increase NO bioavailability. Vitamin D administration in the study of Abeer M. Mahmoud et al. improved flow-induced and acetylcholine-induced dilation of arterioles isolated from adipose and subcutaneous tissues of bariatric patients. The effects have been connected with increased NO production in the resistance arterioles. Achieved improvement in vessels’ reactivity was diminished by eNOS inhibition via NO synthase inhibitor- N(ω)-nitro-L-arginine methyl ester (L-NAME). Interestingly, researchers noted that vitamins D actions were more significant before the bariatric surgery and consequent weight loss [59]. The extent of the therapeutical response to the vitamin D treatment may be related to skin pigmentation. Due to UV ray-absorbing properties, melanin and its relatively higher concentrations can contribute to impaired vitamin D production in adults with darker pigmentation, placing such populations at risk for vitamin D deficiency [60,61,62]. Reduced cutaneous microvascular vasodilation in response to local heating and reduced nitric oxide (NO) contribution to that response have been observed in college-aged African Americans (AA) compared to European American (EA) adults [63,64,65]. It is possible that modulation of NO-mediated signaling can be utilized to limit the discrepancies. Wolf, S. T. et al. reported a mitigation of such differences after 4 weeks of 2000 IU/day oral vitamin D supplementation. They achieved significant improvement in serum 25(OH)D concentrations of AA (from 17.93 ± 5.24 to 26.07 ± 3.73 ng/mL, *p* = 0.04; g = 1.66) but not of EA (*p* = 0.16). Vitamin D supplementation increased the NO contribution to the local heating-induced vessel dilation in AA (from pre- to post-supplementation (29.83 ± 13.70 vs. 46.79 ± 21.93% max, *p* = 0.01; g = 0.89)), abolishing the difference between groups (*p* = 0.47). Its assessment was possible thanks to L-NAME administration and subsequent NO-dependent vasodilation (%NO) quantification [66]. The report of Harris et al. seems to be consistent in terms of vascular effect. Sixteen-week-long supplementation of 60,000 IU monthly oral vitamin D3 (~2000 IU/day) proved to be effective at improving vascular endothelial function in AA adults [67]. Although the observed increase in NO concentrations appears to be statistically significant in the present study, the Spearman r correlation analysis did not show any significant relations between the values of NO and vitamin D concentration after its supplementation. A similar neutral effect on nitric oxide has been observed in PCOS women. The condition makes the suffering prone to multiple metabolic disorders [68,69,70]. A meta-analysis of Akbari et al. concluded that vitamin D supplementation resulted in a significant decrease in hs-CRP, but did not affect NO levels [71].

The second parameter in which significant increase might be observe is AOPP. The difference between the mean concentrations of AOPP before and after treatment (3.87), as shown by the Wilcoxon pair test, was statistically relevant (*p* = 0.047). Characterized for the first time by Witko-Sarsat et al. [72], advanced oxidation protein products (AOPPs) are considered adequate markers of oxidative stress and related tissue injury. Increased AOPP levels have been reported in both obesity and diabetes [73,74]. Koçak et al. demonstrated that serum AOPP levels were significantly elevated in obese and diabetic women compared to healthy control. In all cases, AOPP levels have been positively correlated with blood glucose concentration and age [75]. A simultaneous relative increase in IL-6 levels could be observed, which supports previous findings associating women’s obesity and IL-6 elevation [76,77]. In the study of Oliveira et al. assessing AOPP, they showed a relation between oxidative and nitrosative stresses and vitamin D deficiency in multiple sclerosis patients. Researchers observed decreased levels of AOOP in patients with 25(OH)D concentrations lower than 20 ng/mL than in those with ≥20 ng/mL (133.83 ± 58.95 vs. 164.99 ± 91.40, *p* = 0.046). However, after further analysis, it remained statistically irrelevant [78]. Gradinaru et al. found significantly negative associations between vitamin D status and the susceptibility of LDL to oxidation, the AOPPs, and certain cardiovascular risk biochemical markers like AGEs and nitric oxide metabolic pathway products (NOx) in the elderly, mostly obese patients with IFG and/or T2DM [79]. After correlation analysis in our own study, there was no relation between vitamin D supplementation and post-therapy concentrations of AOPP.

For the first time, a report on a potentially advantageous impact of a sufficient vitamin D level on inflammatory status in humans was presented by Jablonski et al. [80]. They found that vascular endothelial cell expression of the p65 subunit of NFκB, being a major pro-inflammatory nuclear transcription factor, and IL-6, a pro-inflammatory cytokine and downstream target of NFκB, was higher in 25(OH)D deficient patients when compared to middle-aged/older adults who were sufficient in that manner. In the study of our own, measurements of MCP-1, IL-6, PACAP, and CX3CL1 concentrations did not show significant differences before and after vitamin D therapy. Ni et. al., in their research conducted on diabetic rats, demonstrated that vitamin D treatment decreases significantly hepatic expression of pro-inflammatory mediators such as NF-κB and MCP-1 [49]. Wamberg et al., in a series of studies, reported that incubation with 1,25(OH)2D decreased the expression of MCP-1, IL-6, and IL-8 and reduced IL-8 protein secretion in human adipose tissue (AT) fragments [81]. In the follow-up evaluation, supplementation with a daily dose of 7000 IU of vit. D for 26 weeks did not affect the expression of the inflammatory markers in AT or the concentration of circulating inflammatory markers [82]. Similar discrepancies have been observed in numerous studies. On one hand, treatment for 3 years with 700 IU vitamin D plus 500 mg of calcium daily showed no effect of on circulating levels of IL-6 or CRP in healthy adults [83]. Similarly, in the research of Jorde et al. in obese human patients, a 1-year-long treatment with a weekly dose of 40,000 IU of cholecalciferol had no effect on hsCRP, MCP-1, or several other markers of systemic inflammation such as IL-2, IL-4, IL-5, IL-10, IL-12IL-13, IL-17, intercellular adhesion molecule-1, and interferon-gamma [84]. Furthermore, Calton et al. found a lack of benefit with vitamin D supplementation on inflammatory cytokines such as CRP and IL-6 [85]. On the other hand, vitamin D supplementation with 3332 IU daily for 1 year during a weight loss trial of obese subjects enhanced the decrease in TNFα but not CRP or IL-6, compared to placebo [86]. The strongest conclusions regarding the issue can be drawn on the basis of Jamka et al.’s metanalysis, including data from 13 randomized, controlled trials and 1955 overweight and obese subjects [87]. The vitamin D supplementation did not have an influence on CRP (std. mean differences −0.11; 95% CI −0.27–0.04; *p* = 0.15), TNF-α (std. mean differences −0.13; 95% CI −0.38–0.12; *p* = 0.31), and IL-6 concentrations (std. mean differences 0.1; 95% CI −0.43–0.63; *p* = 0.71). In the metanalysis, IL-6 concentrations were evaluated in eight studies. At baseline, in vitamin D groups, the average plasma concentrations of IL-6 varied between 1.00 and 8.90 pg/mL. There was no statistical significance of vitamin D supplementation on plasma concentrations of IL-6 (std. mean differences 0.1; 95% CI −0.43–0.63; *p* = 0.71). The authors brought to attention that reported results might have been affected by differences in ethnicity, advanced age, and sex age [87,88].

Fractalkine is expressed in adipocytes and has been shown to promote monocyte adhesion in adipose tissue from obese patients [89]. It takes part in the regulation of pancreatic islet β cell function and insulin secretion [90]. There is vast evidence that fractalkine/CX3CL1 is actively involved in many states related to inflammation such as atherosclerosis, HIV infection, or cancer [14]. Recent studies prove that serum fractalkine levels may constitute a potent diagnostic marker of childhood onset of SLE, independently of stage of lupus-related nephritis [91]. In the studies of Shinzari et al., subgroups of obese patients presented impaired reactivity to nitric oxide-dependent vasodilator stimuli and enhanced ETA-dependent vasoconstrictor tone when increased circulating fractalkine levels were observed. The authors noted that vascular function impairment may be directly linked to increased plasma concentrations of atherogenic substances including fractalkine [92,93]. Thus, increased LDL-cholesterol or reduced HDL-cholesterol have been reported to elevate fractalkine’s levels. They proposed the presence of lipid abnormalities as a potential key factor in the underlying mechanism of its influence [94,95,96]. In the study of Yegorov et al., 21 cytokines were measured in serum at baseline and after 6 months of vitamin D supplementation in deficient children. Vitamin D deficiency was linked to the reduction of such chemokines as MIP-1α (CCL3) and IL-8 (CXCL8). This relation was not observed with fractalkine. The median blood 25(OH)D concentration at baseline was 13.7 nmol/L (IQR = 10.0–21.7). Supplementation tripled blood 25(OH)D levels (*p* < 0.001) [97]. Another similar trial, conducted by Davaasambuu et al., also in children, showed no statistically significant elevation of various cytokines (IL-1b, 2, 4, 5, 6, 7, 8, 10, 12p70, 13, 17a, 21, 23, GM-CSF, IFN-γ, TNF-α, ITAC, Fractalkine, MIP1a, MIP1b, MIP3a) except the levels of granulocyte–macrophage colony-stimulating factor (GM-CSF) following vitamin D oral supplementation [98].

Unfortunately, data available in literature regarding vitamin’s D influence on PACAP are limited. Nevertheless, PACAP relation with inflammatory processes and obesity-related metabolic changes has been previously described [99,100]. It is expressed both in the peripheral and central nervous systems. The cells of the mediobasal hypothalamus (MBH) contain PACAP in the ventromedial hypothalamic nucleus (VMN), which serves a key role in modulating sympathetic nervous system activation to regulate glucose metabolism, energy expenditure, thermogenesis, and satiety [99]. The study of Green et al. showed that 14 days of treatment with the PACAP receptor antagonist had an unbeneficial effect on glucose tolerance and insulin sensitivity in obese diabetic ob/ob mice [101]. Although details of PACAP’s influence remain unclear, it has been proven that PACAP mediates sympathetic effects of leptin in a tissue-specific manner. The data brought by Tanida et al. suggest that PACAP signaling is connected to leptin’s control of feeding patterns and lipocatabolic sympathetic outflow. What is interesting is that the renal sympathetic traffic appears to be spared and not affected [102].

Despite many reports regarding the abovementioned markers and therapies, the primary outcomes remain the most important issues for clinical practitioners. There are many findings implying the efficacy of vitamin D supplementation in diseases and processes related to inflammation (reviewed in [103]). Levels of vitamin D higher than 50 mmol/L were connected with reduced risk of prostate and colorectal cancer by 30–50% [104,105]. Martineau et al., in their metanalysis, presented evidence on a possible protective effect of vitamin D against acute renal injury [106]. Some recent studies have presented data about differences in vitamin D dosages and their influence on the course of COVID-19 and the risk of developing severe acute respiratory syndrome coronavirus-2 (SARS-CoV-2) [107,108]. Vitamin D deficiency may be related to other inflammatory-based diseases such as allergies. Negative correlation between low concentrations of 25(OH)D and IgE in the serum of studied children have been observed [109]. What is more, antenatal supplementation of vitamin D preparation has been shown to decrease a risk of food allergies in infants with GT/TT genotype [110].

Our study has some potential limitations, within which our findings should be interpreted carefully. First, as in much quantitative research, was a relatively modest sample size and lack of probability sampling due to a strong regional focus of the studied population. Second, body mass composition was determined with the use of an impedance body composition analyzer rather than utilizing techniques based on magnetic resonance, computer tomography, or even near infrared spectroscopy. Although certain inclusion and exclusion criteria were applied, there were factors with the potential impact, such as medications other that hormonal treatment, that did not affect the qualification process. All the above remarks need to be taken into consideration in the follow-up studies in this field, which, hopefully, will be free from restrictions resulting from the pandemic period and related limitations in the collection of data.

## 5. Conclusions

The data presented in this paper throw some light on relations of vitamins D and certain indirect systemic responses related to inflammation. Three months of vitamin D therapy did not induce any statistically significant changes in serum levels of MCP-1, IL-6, CX3CL1, and PACAP. The supplementation was related to a significant increase in measurements of NO and AOPP levels. The correlation analysis between vitamin D concentration after its supplementation and the concentration of molecular parameters did not show any significant relations after the 3-month vitamin D therapy. A summary of information from literature remains inconclusive. Our own findings, in some aspects, are consistent with data already published. On the other hand, a lack of statistical significance or correlation between the vitamin D supplementation and changes observed in levels of the markers may reflect the limitations of the present study, as well as being related to methodological differences between this trial and those mentioned in the discussion. Additional studies are essential to verify the efficacy of vitamin D supplementation on modifying certain inflammatory markers and related clinical consequences. Subsequent moderation of inflammatory processes, vascular reactivity, and free radicals’ creation, if proven to modify certain outcomes, may be utilized to treat obesity-related conditions when concomitant vitamin D deficiency is being diagnosed.

## Figures and Tables

**Table 1:** Obesity classification.

BMI (kg/m^2^)	WHO Classification
<18.5	Underweight
18.5–24.9	Normal weight
25.0–29.9	Overweight
30.0–34.9	Obesity I grade
35.0–39.9	Obesity II grade
>40	Obesity III grade

According to WHO recommendations, 13 patients were classified as obesity grade I, 12 patients as obesity grade II, seven patients as obesity grade III, and one patient as overweight.

**Table 2:** Power of correlation.

Correlation Factor R	Statistical Power of Correlation
0.0–0.3	None
0.3–0.4	Weak
0.4–0.7	Moderate
0.7–0.9	Strong
0.9–1.0	Very strong

**Table 3:** Body weight and BMI values before and after therapy (Wilcoxon’s test).

Parameter	Time Point	N	$\bar{x}$	SD	Min	Max	*p*-Value
Body mass	Time point 0 (before)	33	110.50	23.91	75.9	194.5	0.089
	Time point 1 (after)	33	110.24	25.45	75.7	195.2	
BMI	Time point 0 (before)	33	36.92	6.00	26.6	55.0	0.153
	Time point 1 (after)	33	36.82	6.48	27.0	55.2	

**Table 4:** Descriptive and statistical analyses of the comparison of skeletal muscle mass, adipose tissue mass, % of adipose tissue, and the level of visceral fat before and after the 3-month vitamin D supplementation (Wilcoxon’s test).

Parameter	Time Point	N	$\bar{x}$	SD	Min	Max	*p*-Value
Skeletal muscles mass (kg)	Time point 0 (before)	33	37.18	8.34	24.6	65.5	0.437
	Time point 1 (after)	33	36.88	9.20	24.3	66.8	
Adipose tissue mass (kg)	Time point 0 (before)	33	45.19	13.31	30.0	81.3	0.964
	Time point 1 (after)	33	45.87	13.80	30.4	82.7	
% Adipose tissue	Time point 0 (before)	33	40.81	5.98	29.7	53.2	0.297
	Time point 1 (after)	33	41.42	6.53	28.4	52.9	
Visceral fat levels	Time point 0 (before)	33	17.52	2.68	11.0	20.0	0.583
	Time point 1 (after)	33	17.45	2.87	11.0	20.0	

**Table 5:** Body mass composition and its changes during the therapy in women.

Women (*n* = 16)		Body Mass (kg)	BMI (kg/m^2^)	Skeleton Muscles Mass (kg)	Fat Tissue Mass (kg)	% Fat Tissue	Visceral Fat Tissue
**Before Therapy**	Minimum	75.90	26.60	24.60	30.00	35.40	11.00
	Maximum	147.8	51.10	40.40	78.60	53.20	20.00
	Mean	101.0	35.92	31.73	45.23	44.48	16.81
	Std. Deviation	19.07	6.103	5.241	13.56	5.795	3.124
	Std. Error of Mean	4.768	1.526	1.310	3.389	1.449	0.7811
**After Therapy**	Minimum	75.70	27	24.30	30.70	35.70	11
	Maximum	150.4	52	40.10	79.60	52.90	20
	Mean	100.1	35.60	31.06	45.92	45.26	17.06
	Std. Deviation	19.34	6.326	5.183	13.45	5.353	3.08
	Std. Error of Mean	4.836	1.582	1.296	3.361	1.338	0.77

**Table 6:** Body mass composition and its changes during the therapy in men.

Men (*n* = 17)		Body Mass (kg)	BMI (kg/m^2^)	Skeleton Muscles Mass (kg)	Fat Tissue Mass (kg)	% Fat Tissue	Visceral Fat Tissue
**Before Therapy**	Minimum	101.7	31.10	34.40	31.6	29.70	14
	Maximum	194.5	55	65.50	81.3	44.70	20
	Mean	119.4	37.85	42.32	45.15	37.36	18.18
	Std. Deviation	25.06	5.923	7.458	13.48	3.748	2.069
	Std. Error of Mean	6.077	1.437	1.809	3.27	0.909	0.501
**After Therapy**	Minimum	97.60	30.40	29.90	30.4	28.40	12
	Maximum	195.2	55.20	66.80	82.70	47	20
	Mean	119.8	37.98	42.35	45.82	37.81	17.82
	Std. Deviation	27.30	6.595	8.849	14.54	5.45	2.698
	Std. Error of Mean	6.621	1.6	2.146	3.526	1.322	0.654

**Table 7:** Descriptive and statistical analyses of the Vitamin D concentration before and after the 3-month supplementation.

Parameter	Time Point	N	$\bar{x}$	SEM	Min	Max	*p*-Value
Vitamin D 25-(OH) (ng/mL)	Time point 0 (before)	33	18.22	1.106	81	28.4	<0.001
	Time point 1 (after)	33	29.89	1.160	19.1	52.1	

**Table 8:** Molecular markers of inflammation: descriptive and statistical analyses (Wilcoxon’s test).

Parameter	Time Point	N	$\bar{x}$	SEM	Min	Max	*p*-Value
MCP1 (ng/mL)	Time point 0 (before)	33	230.35	47.83	10.0	670.0	0.157
	Time point 1 (after)	33	246.41	47.80	10.0	669.0	
IL-6 (pg/mL)	Time point 0 (before)	33	29.67	18.68	2.3	625.9	0.198
	Time point 1 (after)	33	36.39	24.92	2.4	832.7	
CX3CL1 fraktalin (ng/mL)	Time point 0 (before)	33	10.22	0.64	4.0	15.9	0.056
	Time point 1 (after)	33	10.80	0.61	3.4	15.1	
AOPP (nmol/mL)	Time point 0 (before)	33	55.07	12.21	2.5	167.5	0.047
	Time point 1 (after)	33	58.94	12.10	2.5	167.2	
NO (µmoL/L)	Time point 0 (before)	33	39.19	10.96	5.0	39.19	0.021
	Time point 1 (after)	33	7002	13.80	24.2	70.02	
PACAP (ng/mL)	Time point 0 (before)	33	2.30	0.47	0.1	6.5	0.218
	Time point 1 (afteR)	33	2.25	0.48	0.1	6.7	

**Table 9:** Correlation analysis between vitamin D supplementation and pre-therapy concentrations of biomarkers.

Variables	N	R	95% Confidence Interval	*p*-Value
Vitamin D (Before) vs. MCP1 (Before) (ng/mL)	33	0.21	−0.15 to 0.52	0.23
Vitamin D (Before) vs. IL-6 (Before) (pg/mL)	33	0.01	−0.34 to 0.36	0.93
Vitamin D (Before) vs. NO (Before) (µmoL/L)	33	0.13	−0.23 to 0.46	0.44
Vitamin D (Before) vs. CX3CL1 (Before) (ng/mL)	33	−0.03	−0.38 to 0.31	0.83
Vitamin D (Before) vs. AOPP (Before) (nmol/mL)	33	0.235	−0.12 to 0.54	0.18
Vitamin D (Before) vs. PACAP (Before) (ng/mL)	33	0.151	−0.21 to 0.47	0.40

**Table 10:** Correlation analysis between vitamin D supplementation and post-therapy concentrations of biomarkers.

Variables	N	R	95% Confidence Interval	*p*-Value
Vitamin D (After) vs. MCP1 After (ng/mL)	33	0.052	−0.16 to 0.52	0.25
Vitamin D (After) vs. IL-6 After (pg/mL)	33	0.104	−0.40 to 0.30	0.75
Vitamin D (After) vs. NO After (µmoL/L)	33	−0.183	−0.02 to 0.61	0.06
Vitamin D (After) vs. CX3CL1 After (ng/mL)	33	−0.148	−0.08 to 0.57	0.12
Vitamin D (After) vs. AOPP After (nmol/mL)	33	0.156	−0.11 to 0.55	0.16
Vitamin D (After) vs. PACAP After (ng/mL)	33	−0.106	−0.15 to 0.52	0.23

## Data Availability

The data presented in this study are available on request from the corresponding author.
